# Comparative analysis of replication characteristics of BoHV-1 subtypes in bovine respiratory and genital mucosa explants: a phylogenetic enlightenment

**DOI:** 10.1186/1297-9716-42-33

**Published:** 2011-02-15

**Authors:** Lennert Steukers, Annelies P Vandekerckhove, Wim Van den Broeck, Sarah Glorieux, Hans J Nauwynck

**Affiliations:** 1 Laboratory of Virology, Department of Virology, Parasitology and Immunology, Faculty of Veterinary Medicine, Ghent University, Salisburylaan 133, B-9820 Merelbeke, Belgium; 2 Department of Morphology, Faculty of Veterinary Medicine, Ghent University, Salisburylaan 133, B-9820 Merelbeke, Belgium

## Abstract

In general, members of the *Alphaherpesvirinae *use the epithelium of the upper respiratory and/or genital tract as a preferential site for primary replication. Bovine herpesvirus type 1 (BoHV-1) may replicate at both sites and cause two major clinical entities designated as infectious bovine rhinotracheitis (IBR) and infectious pustular vulvovaginitis/balanoposthitis (IPV/IPB) in cattle. It has been hypothesized that subtype 1.1 invades preferentially the upper respiratory mucosa whereas subtype 1.2 favors replication at the peripheral genital tract. However, some studies are in contrast with this hypothesis. A thorough study of primary replication at both mucosae could elucidate whether or not different BoHV-1 subtypes show differences in mucosa tropism. We established bovine respiratory and genital organ cultures with emphasis on maintenance of tissue morphology and viability during in vitro culture. In a next step, bovine respiratory and genital mucosa explants of the same animals were inoculated with several BoHV-1 subtypes. A quantitative analysis of viral invasion in the mucosa was performed at 0 h, 24 h, 48 h and 72 h post inoculation (pi) by measuring plaque latitude and penetration depth underneath the basement membrane. All BoHV-1 subtypes exhibited a more profound invasion capacity in respiratory tissue compared to that in genital tissue at 24 h pi. However, at 24 h pi plaque latitude was found to be larger in genital tissue compared to respiratory tissue and this for all subtypes. These similar findings among the different subtypes take the edge off the belief of the existence of specific mucosa tropisms of different BoHV-1 subtypes.

## Introduction

Alphaherpesviruses in general have a broad epithelial tropism. BoHV-1, the known etiological agent of infectious bovine rhinotracheitis (IBR) and infectious pustular vulvovaginitis/balanoposthitis (IPV/IPB) in cattle, may replicate in both respiratory and genital mucosa. However, respiratory and genital infections have been assigned to different BoHV-1 strains in the past [1].

Although there is proof of the existence of the virus as early as 1941, BoHV-1 was first isolated in 1955 [2,3]. In the late 1950's both the respiratory disease prototype strain Cooper and the genital disease prototype strain K22 were isolated from field cases of respectively IBR and IPV in the United States [4,5]. Importantly, before 1977, a continental discrepancy was present. Genital infections were predominant throughout Europe whereas respiratory infections were mainly prevalent at feedlots in the United States and Canada. Some authors believe that the virulent respiratory strains emerged out of the less virulent genital strains. They state that the enhanced virulence of the virus for the respiratory epithelium is a consequence of rapid passages of the virus in crowded susceptible populations present in "feedlots", typically for the United States at that time. Since 1977, severe "North American like" IBR has emerged on the European continent [6-8].

An attempt was made to see if BoHV-1 could be subdivided into distinct types with different tropisms i.e. whether a correlation could be found between IBR and IPV on the one hand and distinct virus subtypes on the other hand. A classification was made of different BoHV-1 subtypes using restriction endonuclease digestion and reactivity tests on a panel of monoclonal antibodies. BoHV-1.1 was associated with respiratory disease and abortion whereas BoHV-1.2 was regarded as a genital type [9-11]. At that time point distinguished subtype BoHV-1.3 was renamed into BoHV-5 [12,13]. Furthermore, a distinction was made between different BoHV-1.2 subtypes. BoHV-1.2b causes local genital lesions and possibly mild respiratory illness; BoHV-1.2a seems to have both tropism for the genital and respiratory mucosa and is associated with abortion [9,10,14]. However, this postulation about several subtypes possessing diverse mucosa tropisms has been contested since several studies showed no correlation between the different genotypes and their clinical manifestations [6,7,14-18].

A good way to elucidate the relationship between the distinct viral BoHV-1 subtypes and the clinical entities would be to test isolates representing the different subtypes on similar groups of animals under identical conditions. Practical, ethical and financial reasons make this in vivo approach difficult to perform. For that purpose, suitable in vitro systems resembling the in vivo situation and implementing the three R's principle of Russell and Burch (1959), are needed to study primary host-virus interactions.

The aim of the present study was to evaluate quantitatively the replication characteristics of different BoHV-1 subtypes in in vitro systems of bovine respiratory and genital mucosa explants. Previously, we optimized an in vitro bovine respiratory organ culture [19]. Here, we elaborate the set up of an air-liquid interface bovine genital organ culture using vestibulum vaginae tissue. Similarly as for the previously optimized respiratory organ culture, the effect of an in vitro culture on viability and morphometry was extensively evaluated up to 96 h of in vitro cultivation for the genital organ culture. Furthermore, bovine organ cultures of trachea and vestibulum vaginae derived from the same animals were infected with several BoHV-1.1, BoHV-1.2a and BoHV-1.2b isolates. A quantitative analysis of viral mucosal invasion was performed for the different BoHV-1 subtypes and compared at 0 h, 24 h, 48 h and 72 h post inoculation (pi) by measuring plaque latitude and penetration depth underneath the basement membrane (BM).

## Materials and methods

### Selection of animals

At the slaughterhouse, seven different female slaughter animals between 3 and 5 years old were selected. Criteria to include the animals in this experiment were based on both female reproductive hormone level and BoHV-1 specific serological status. At slaughter, a thorough palpation and visual inspection of the ovaries was performed to select cows with a clear marked corpus luteum. Moreover, blood was collected at slaughter. On all sera, a progesterone determination and a complement-dependent seroneutralization (SN)-test were performed to determine peripheral blood progesterone levels and BoHV-1 specific antibody titers respectively.

### Gathering of tissues and preparation of air-liquid interface organ cultures

From seven different cows, proximal trachea and vestibulum vaginae were collected at the local abattoir immediately after slaughter. A similar set up was used for bovine respiratory and genital mucosal explants. Briefly, tissues were immediately placed in phosphate buffered saline (PBS), supplemented with 1 μg/mL gentamycin (Invitrogen, Paisley, UK), 1 mg/mL streptomycin (Certa, Braine l'Alleud, Belgium), 1 mg/mL kanamycin (Sigma, St. Louis, MO, USA), 1000 U/mL penicillin (Continental Pharma, Puurs, Belgium) and 5 μg/mL fungizone (Bristol-Myers Squibb, New York, USA) for transportation to the laboratory. Respiratory and genital mucosa was stripped from the underlying layers. Small square tissue pieces were made and placed on fine-meshed gauze for culture. The explants were cultivated in serum-free medium (50% DMEM (Invitrogen)/50% Ham's F-12 GlutaMAX (Invitrogen)) supplemented with 0.1 mg/mL streptomycin (Certa), 100 U/mL penicillin (Continental Pharma) and 1 μg/mL gentamycin (Invitrogen) for up to 96 h (37 °C, 5% CO_2_). Genital tissue from four animals was used for an extensive morphometry and viability analysis. Both respiratory and genital tissue of three other cows was used to evaluate BoHV-1 subtype replication characteristics.

### Evaluation of tissue viability

The effect of in vitro cultivation on viability was evaluated using an In Situ Cell Death Detection Kit (Roche Diagnostics Corporation, Basel, Switzerland), based on Terminal deoxynucleotidyl transferase mediated dUTP Nick End labeling (TUNEL). The test was performed according to the manufacturer's guidelines. The amount of TUNEL-positive cells was counted from five randomly chosen fields of 100 cells in both epithelium as well as lamina propria. An analysis was made at 0 h, 24 h, 48 h, 72 h and 96 h of cultivation.

### Evaluation of tissue morphometry

At 0 h, 24 h, 48 h, 72 h and 96 h of cultivation, explants of genital tissue were gathered for further analysis. A thorough morphometrical assessment was performed by means of light microscopy, transmission electron microscopy and scanning electron microscopy in an analogous way as for the previously optimized bovine respiratory organ culture.

#### Light microscopy

Fixation of explants was performed at the different time points by submerging them in a phosphate-buffered 3.5% formaldehyde solution for 24 h. Embedding in paraffin and further processing happened according to standard methods. As a parameter for the effect of in vitro culture on the epithelial morphometry, epithelial thickness was measured by means of a haematoxylin-eosin staining. At 40×magnification, five randomly selected places in five randomly chosen fields were measured in each explant. Next, a reticulin staining to evaluate continuity and thickness of the basement membrane was carried out. Five randomly chosen places in five randomly chosen zones were measured in each sample. Finally, the structure of the connective tissue was evaluated by means of a Van Gieson staining. In five randomly chosen fields, the relative amounts of collagen and nuclei were calculated in a defined region of interest (ROI) by setting a threshold. All measurements and calculations were performed using the Cell F Software linked to a BX61 light microscope (Olympus, Hamburg, Germany) (magnification 40×).

#### Scanning electron microscopy

After gathering the explants at the defined time points, fixation of the explants in a HEPES-buffer containing 2% paraformaldehyde and 2.5% glutaraldehyde solution during 24 h was followed by a post-fixation step in an un-buffered 1% osmium tetroxide solution for 2 h. Then, the fixed explants were dehydrated through ascending grades of alcohol and critical point dried with CO_2 _(CPD 030, Balzers, Sercolab, Merksem, Belgium), mounted on metal stubs, platinum-coated (JFC-1300 Autofine Coater, Jeol, Tokyo, Japan) and examined by a Jeol JSM 5600 LV scanning electron microscope (Jeol). The integrity of the epithelium was examined using 1 500× and 5 000× magnification.

#### Transmission electron microscopy

After an overnight fixation step at 4 °C in Karnovsky's fixative (2% paraformaldehyde and 2.5% glutaraldehyde in 0.2 M sodium cacodylate buffer pH 7.4), the explants were rinsed in 0.1 M sodium cacodylate buffer pH 7.4 for 8 h. Next, an overnight post-fixation step in 2% osmium tetroxide at 4 °C was performed. Further, the tissues were dehydrated stepwise in ascending grades of alcohol before they were embedded in a low viscosity embedding (LVR) medium (Agar Scientific ltd., Stansted, Essex, UK). Finally, ultrathin sections of embedded material were cut, using a diamond knife on an Ultramicrotome Ultracut EM UC6 (Leica Microsystems, Wetzlar, Germany). Afterwards, a Leica Microsystems EM staining was performed and samples were analyzed on a JEM-1010 transmission electron microscope (Jeol) operating at 60 kV.

### Inoculation of explants with different BoHV-1 subtypes

Different strains were used, representing the known different genotypes of BoHV-1. The Cooper strain, which is considered as the prototype 1.1 subtype, was isolated in Colorado [5]. Another 1.1 strain, Lam, was isolated in 1972 from an IBR-case in the Netherlands [20,21]. The prototype 1.2 strain K22 is typed as a subtype 1.2b [9]. This isolate was obtained from an IPV outbreak in New York [4]. The Schönböken isolate is designated as a BoHV-1.2a subtype and was isolated in Germany [22,23]. All strains obtained were from unknown passages. From each of them, a second passage was produced and utilized in our laboratory.

After 24 h of cultivation, explants were inoculated with the different strains. The explants were taken from their gauze and placed in a 24-well plate after rinsing with warm medium. In each well, 1 mL of virus-containing medium (10^7 ^TCID_50_/mL) was added. The submerged explants were incubated for 1 h (37 °C, 5% CO_2_). Before the tissues were placed back again on their gauze, they were thoroughly washed. In that way, different explants, both proximal trachea and vestibulum vaginae from the same animal were infected with the different strains. Samples were collected at 0 h, 24 h, 48 h and 72 h pi. At every time point, 2 respiratory and 2 genital explants from each animal for each strain were collected. Finally, all gathered explants were embedded in a cryoprotection medium (Methocel^®^, Fluka (Sigma)) and frozen at -70 °C.

### Evaluation of BoHV-1 subtype kinetics

From all collected samples, cryosections were produced, fixed in methanol (-20 °C, 100%) and kept at -20 °C until staining. An immunofluorescence staining was performed to evaluate plaque latitude and plaque penetration depth underneath the BM. Firstly, to stain the BM, mouse anti-collagen VII antibodies (Sigma) and goat anti-mouse Texas Red^® ^(Molecular Probes (Invitrogen)) were used. Next, an FITC^®^-labeled goat anti-IBR polyclonal antiserum (VMRD, Pullman, WA, USA) directed against viral proteins was applied. Mounted samples were analyzed by means of a confocal microscope (Leica TCS SP2 confocal microscope). Using the software program ImageJ, dissemination characteristics were monitored.

### Statistical analysis

The data obtained were assessed using SPSS software (ANOVA) to evaluate the variance. The results shown represent means + standard deviation of quadruple and triple independent experiments of respectively viability/morphometry analysis and analysis of viral subtype replication characteristics. The results with *P *values of ≤ 0.05 were considered significant.

## Results

### Progesterone determination and Sn-test

All animals included in the experiment had a peripheral blood progesterone (P4) level of > 1 ng/mL, suggesting they were in the luteal phase of the reproductive cycle. Three animals showing an Sn-titer of < 2 for BoHV-1 specific antibodies were selected for the study of BoHV-1 subtype dissemination characteristics. The four other animals were used for a thorough morphometry and viability analysis. The latter animals had an Sn-titer ranging from 2 to 96.

### Tissue viability

We saw no major differences in the occurrence of apoptosis in the epithelium with increasing times of in vitro culture. However, a small significant increase in apoptotic epithelial cells was observed at 96 h of cultivation. For the connective tissue, we noticed a small increase in the occurrence of apoptosis during culture time (to 7.7 ± 2.7 at 96 h). Values of the effect of in vitro culture on the viability of bovine genital mucosa explants are given in Table 1.

**Table 1:** Percentage of TUNEL-positive cells in epithelium and lamina propria as a parameter for the effect of in vitro culture on the viability of bovine genital mucosa explants.

		% of TUNEL-positive cells at ... h of cultivation				
		
		0	24	48	72	96
Vestibulum Vaginae	Epithelium	0.3 ± 0.1	0.4 ± 0.3	0.8 ± 0.4	0.3 ± 0.2	1.5 ± 0.5
	Lamina propria	0.9 ± 0.4	3.2 ± 1.5	4.8 ± 3.2	5.8 ± 4.5	7.7 ± 2.7

Values are given as means ± SD

### Morphometry of the epithelium

#### Light microscopy

**Figure 1:**
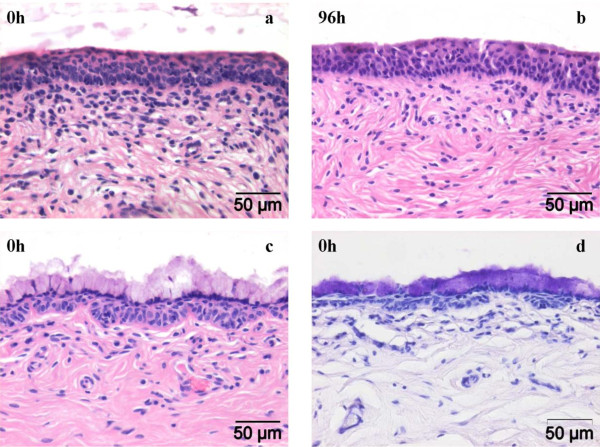
**Photomicrographs of bovine stratified squamous epithelium lining the vestibulum vaginae at 0 h (a) and 96 h (b) of in vitro cultivation (end of experiment) (HE-staining)**. One cow showed zones containing an active mucus-secreting (PAS +) stratified columnar to cuboidal epithelium, prominent at 0 h of in vitro cultivation. This active epithelium is shown in c (HE-staining) and d (PAS-staining).

No significant changes were noticed in the epithelial thickness of the vestibulum vaginae measured during cultivation. A clear non-keratinized stratified squamous epithelium was visible for all samples (Figure 1a-b). However, for all cows, a few zones containing a stratified columnar to stratified cuboidal epithelium were observed. Moreover, one cow (1.03 ng/mL P4 level) showed especially at 0 h of cultivation some columnar to cuboidal cells in the stratified columnar to cuboidal epithelial zones containing a strong periodic acid + Schiff (PAS) positive substance (Figure 1c-d).

#### Scanning electron microscopy

**Figure 2:**
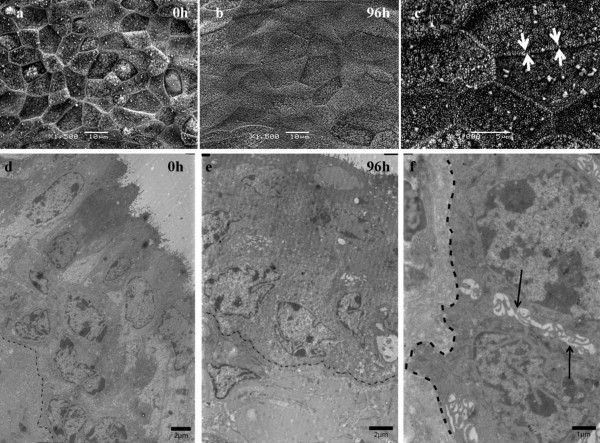
**Scanning electron and transmission electron microscopical images of bovine vaginal epithelium**. Pavement-like cells containing microvilli were seen at all time points (0 h-96 h) of in vitro culture when evaluating the epithelial surface by means of scanning electron microscopy (a-b). Cells are aligned with clear microridges (c, indicated by white arrows). Epithelial integrity and structure was maintained at all time points (0 h-96 h) as seen with transmission electron microscopy (d-e). Starting from 0 h of cultivation, small intercellular spaces between basal cells were observed (f, indicated by black arrows). The dotted line represents the basement membrane (BM).

The evaluation of epithelial integrity by scanning the surface of the epithelium showed no significant changes in epithelial morphology during in vitro culture for up to 96 h. Surface cells had an irregular pavement-like appearance (Figure 2a-b). Moreover, on the surface secretory blebs were visible. All epithelial cells contained stubby microvilli and at the cell borders, clear microridges were visible (Figure 2c). These cell surface structures could be maintained at all time points.

#### Transmission electron microscopy

Overall integrity was evaluated by means of transmission electron microscopy. The epithelial structure was maintained during the entire cultivation period (96 h) (Figure 2d-e). Starting from 0 h of cultivation, small intercellular spaces were observed between some basal cells (Figure 2f). Remarkably, large apical electron-lucent cells randomly spread across the epithelium, were observed starting from time point 0 h of cultivation. Conservation of microvilli at 0 h, 24 h, 48 h, 72 h and 96 h of cultivation was noticed.

### Morphometry of the basement membrane

#### Light microscopy

**Figure 3:**
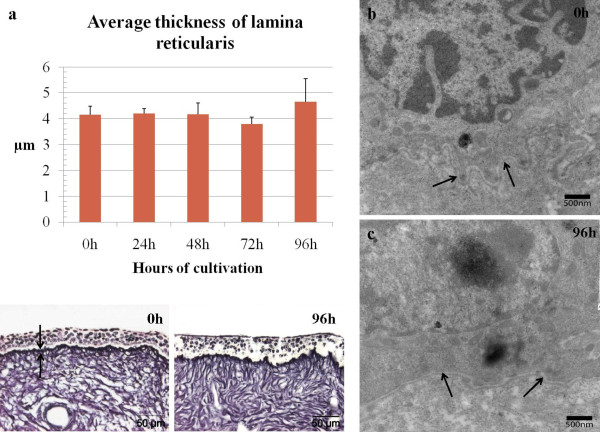
**Evaluation of basement membrane (lamina densa and lamina reticularis) continuity and thickness by means of transmission electron microscopy and light microscopy (reticulin staining)**. Average thickness of the lamina reticularis was monitored throughout in vitro culture (a, black arrows indicate lamina reticularis). No significant changes were observed in lamina reticularis thickness when analyzing reticulin stainings. The lamina densa remained continuous at all time during the entire cultivation period (up to 96 h) as shown by the transmission electron microscopical images (b-c, black arrows indicate lamina densa). Data are represented as means + SD (error bars).

After analysis of the reticulin stained sections, significant changes were not observed regarding the thickness of the lamina reticularis during in vitro culture (Figure 3a).

#### Transmission electron microscopy

The continuity and integrity of the lamina densa of the basement membrane were evaluated by means of transmission electron microscopy. During the 96 h of in vitro cultivation, no significant changes in lamina densa continuity and integrity were found (Figure 3b-c).

### Morphometry of the connective tissue

**Figure 4:**
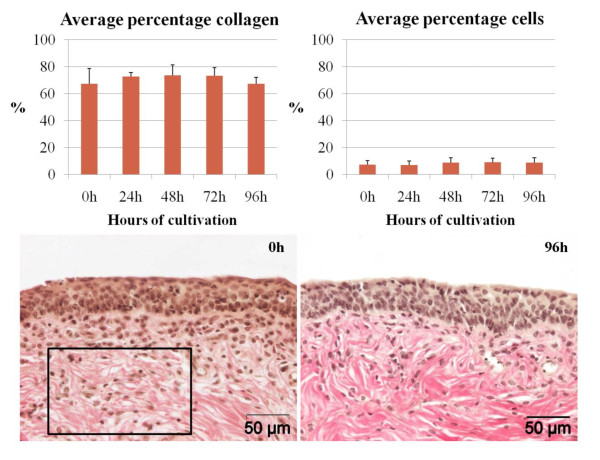
**Average percentages of collagen and cells were measured in the connective tissue and assessed on conservation throughout cultivation (up to 96 h) using Van Gieson stainings**. By giving different colors to collagen and nuclei (setting a threshold), relative amounts of collagen and nuclei were measured within a region of interested (roi) (roi is indicated by a rectangle). Representation of the data is visualized as means + SD (error bars).

Using a Van Gieson staining, no significant changes were noticed in relative percentage of collagen and nuclei in the connective tissue with increasing time after sampling (Figure 4).

### BoHV-1 subtype kinetics

**Figure 5:**
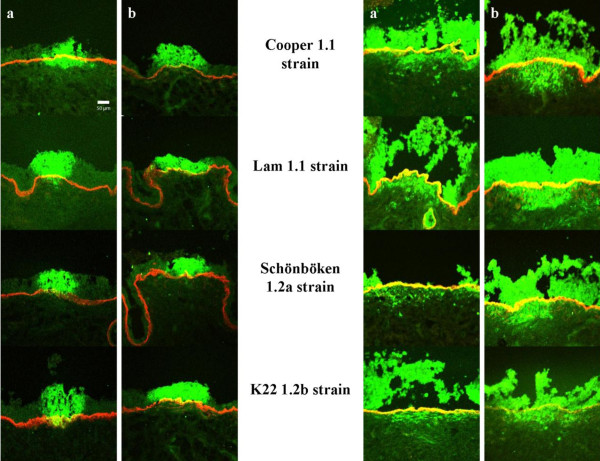
**Confocal fluorescent images of bovine respiratory (a) and genital (b) mucosa explants inoculated with different BoHV-1 subtypes at 24 h pi (left side) and 72 h pi (right side)**. Viral antigen is colored with an FITC^®^- labeled goat anti-IBR polyclonal antiserum. Collagen VII is marked with mouse anti-collagen VII and goat anti-mouse Texas Red^® ^antibodies.

Individual plaques were visible starting from 24 h pi for all strains in all tissues. All different BoHV-1 subtypes were found to spread in a plaquewise manner in both the respiratory and genital mucosa derived from three animals. The analysis of plaque latitude and penetration depth underneath the BM was performed at different time points pi for all BoHV-1 subtypes on proximal trachea and vestibulum vaginae (Figure 5).

#### Plaque latitude

**Figure 6:**
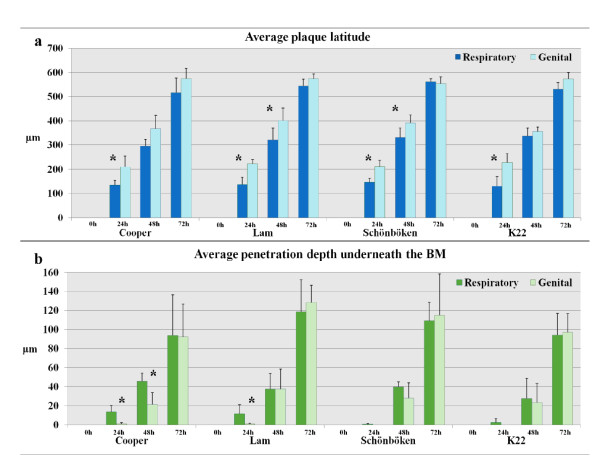
**Replication characteristics of different BoHV-1 subtypes in respiratory and genital tissues derived from the same animals**. Evolution of plaque latitude (a) and plaque penetration depth underneath the basement membrane (BM) (b) was evaluated at 0 h, 24 h, 48 h and 72 h pi. Data are given as means + SD (error bars). Significant differences between respiratory and genital tissue are indicated by means of asterisks.

Individual plaque measurement was performed at 0 h, 24 h, 48 h and 72 h pi (Figure 6a).

##### In relation to time

At 0 h pi, no viral plaques were visible. A significant increase in plaque latitude was observed between 0 h, 24 h, 48 h and 72 h for all strains on the different tissues.

##### In relation to tissue

Interestingly, we observed a significant difference in plaque latitude between proximal trachea and vestibulum vaginae at 24 h pi. This was noticed for all different BoHV-1 subtypes. Lateral spread was more clear in vestibulum vaginae compared to the proximal trachea. At 48 h pi, the Lam and Schönböken strain still exhibited a higher dissemination capacity in the epithelium of the vagina compared to the trachea whereas Cooper and K22 did not. All strains no longer showed a significant difference in plaque latitude when comparing proximal trachea and vestibulum vaginae at 72 h pi (Figure 6a).

##### In relation to strain

When comparing the strains, no significant differences between the subtypes could be observed at 0 h, 24 h, 48 h and 72 h pi. All did exhibit a higher average plaque latitude at 24 h pi in vestibulum vaginae compared to proximal trachea (Figures 5-6a).

#### Plaque penetration depth underneath the BM

The average invasion depth of different BoHV-1 subtypes was measured at 0 h, 24 h, 48 h and 72 h pi (Figure 6b).

##### In relation to time

At 0 h pi, no vertical spread was observed for all different BoHV-1 subtypes in both proximal trachea and vestibulum vaginae. All strains included showed an increase in plaque penetration depth between 0 h, 24 h, 48 h and 72 h pi in proximal trachea and vestibulum vaginae. This increase was significant for all strains on all tissues between 48 h and 72 h pi.

##### In relation to tissue

Both respiratory strains Cooper and Lam showed a clear advantage on proximal trachea compared to the vestibulum vaginae. The average invasion depth was significantly higher in respiratory tissue than in genital tissue and this at 24 h (Lam en Cooper) and 48 h (Cooper) pi. Interestingly, for both designated genital 1.2-strains Schönböken and K22, we did not see a significant difference when comparing both target tissues. However, we must mention that for both genital strains at 24 h pi, not one plaque crossed the BM in the genital tract whereas some plaques did in the respiratory tract. At 48 h (except Cooper) and 72 h pi, no significant differences were found when comparing all strains on proximal trachea and vestibulum vaginae (Figures 5-6b).

##### In relation to strain

We observed no significant differences amongst strains at 0 h, 24 h, 48 h and 72 h pi. The penetration depth of the Cooper strain in the proximal trachea was significantly higher than the penetration depth of the Schönböken strain in the proximal trachea only at 24 h pi.

## Discussion

In this study, we developed and utilized bovine respiratory and genital organ culture systems to reconstruct key elements in BoHV-1 mucosal invasion and moreover, to shed some light on the current classification of different BoHV-1 subtypes with particular tropisms. Because of their in vivo relevance, these in vitro models are highly appreciated since laboratory animal use is diminished and confounding factors such as individual animal variation and environmental factors are excluded. Therein lies their strength, when comparing an array of strains/subtypes on viral behavior at mucosal entry ports. Until now, organ cultures of bovine vestibulum vaginae have not been described.

The first part of the current study consisted of the development of an organ culture of bovine vestibulum vaginae. Some interesting facts were seen when evaluating tissue morphology. One cow showed highly active columnar to cuboidal cells in the stratified columnar to cuboidal epithelial zones containing a PAS positive substance. It is noteworthy that at the time of sampling, this cow was in a transition phase of the reproductive cycle namely from a progesterone to an estrogen dominance or vice versa (P4 1.03 ng/mL), this mucin production might be an outcome of estrogen influence on the epithelium [24]. These hormone-related findings are important since herpesvirus infections at the genital site are known to be influenced by reproductive hormone levels [25]. Starting from 0 h of cultivation, few intercellular spaces were visible between basal cells of the epithelium. We made the same observations when establishing the bovine respiratory mucosa explant model [19]. When using transmission electron microscopy, salient large electron-lucent apical cells, randomly spread across the epithelium starting from 0 h of cultivation, were noticed. Regli and Kress saw similar cells in the vaginal epithelium of the marsupial *Monodelphis domestica*, describing the remarkable fact as cells damaged by the previous desquamation process or not yet differentiated cells [26]. However, no significant changes in the number of these particular cells were found throughout cultivation. At 96 h of cultivation, we observed a small increase in the number of apoptotic epithelial cells together with an increase in apoptotic lamina propria cells as culture time passed by. An analogous trend was seen in the viability assessment of upper respiratory tract cultured tissues [19]. Overall, we can state that the newly developed organ culture of bovine vestibulum vaginae was successfully maintained for at least 96 h in culture at air-liquid interface without demonstrable changes in tissue architecture or viability.

In the second part of the study, bovine respiratory and genital mucosa explants from the same animals were inoculated with several BoHV-1 subtypes. We clearly saw that both respiratory and genital mucosa are susceptible to infection with all different BoHV-1 subtypes. This dual tropism is also seen in closely related animal alphaherpesviruses with either emphasis on the genital tract such as Caprine herpesvirus type 1 and Cervid herpesvirus type 2; or on the respiratory tract such as Cervid herpesvirus type 1 and Suid herpesvirus type 1 (PrV) [27-31]. However, subdivision into different subtypes of the latter viruses never was an issue. Moreover, comparison of the DNA sequences of different BoHV-1 subtypes is generally accepted to show at least 95% homology [32,33]. This percentage is analogous among some strains of other herpesviruses such as PrV. Pair wise analysis of full genome DNA sequence comparisons of different strains of PrV has been performed before [34]; some strains did exhibit a degree of divergence of around 5%, which is similar when comparing BoHV-1 subtypes 1.1 and 1.2.

Next, a thorough analysis on the dissemination characteristics of different BoHV-1 subtypes on both respiratory and genital mucosa divulged important information on the current phylogenetic classification. Looking at average plaque penetration depth, key findings were twofold. Firstly, the so called respiratory subtypes Cooper and Lam invaded significantly deeper in respiratory tissue compared to genital tissue at 24 h and 48 h pi. However, secondly, in general all BoHV-1 subtypes exhibited a more profound invasion capacity in respiratory tissue compared to that in genital tissue at 24 h pi. It is known that massive accumulation of neutrophils and activation of macrophages reach peaks between 24-48 h post BoHV-1 infection [35]. Thus, the latter findings clearly demonstrate the outright advantage of all subtypes when invading respiratory tissues in pathogenesis. Caprine herpesvirus type 1, which is responsible for genital and respiratory disease in goats, shows a similar advantage on the respiratory tract. In general, infection by the genital route is often locally confined while the virus can spread by viremia after respiratory infection [30]. The observed rapid spread through the BM towards blood vessels in respiratory tissues and the avoidance of the local immunity peak, might be an explanation why BoHV-1-induced abortion is mainly seen after a respiratory infection [1]. Concerning average plaque latitude, all strains replicated to a similar extent in epithelial cells. It is noteworthy that at 24 h pi, plaque latitude was found to be higher in genital tissue compared to respiratory tissue and this for all subtypes. This higher plaque latitude in the genital tract may be a compensation of the virus for the hampered spread into the depth compared to respiratory tissue, as mentioned above. Differences in BoHV-1 spread between upper respiratory and genital tissues could potentially be related to differences in apoptosis during cultivation of these different tissues. The only differences observed in BoHV-1 subtype replication characteristics in the different tissues were noticed at 24 h pi. However, at this point (48 h of cultivation), we see very little, if any, differences in the occurrence of apoptosis between the upper respiratory and genital tract. For this, we conclude that the small increase in apoptotic cells throughout cultivation is likely negligible for the replication characteristics of BoHV-1 strains.

Our findings are in line with the vision of McKercher. He believes that virulent respiratory strains arose out of the less virulent genital strains and that in general the virus may be limited in invasion capacity due to biochemical and physiological characteristics of the vagina with emphasis on barrier function [6,7].

We can conclude that the respiratory and genital organ culture is suitable to study BoHV-1 invasion at primary entry ports and that they can be extrapolated to other species and viruses. Taken together, our findings and the existing knowledge on BoHV-1 classification, clearly take the edge off the belief of the existence of specific mucosa tropisms of different BoHV-1 subtypes and make the phylogenetic BoHV-1 subdivision rather farfetched. The question arises whether this could be a difference in virulence between strains, as seen in all other viruses, rather than a difference in mucosa tropism?

## Competing interests

The authors declare that they have no competing interests.

## Authors' contributions

LS set up the study design, carried out the optimization of the organ culture as well as the infection experiments and the processing of all samples, performed the statistical analysis and drafted the manuscript. APV assisted in the set up of both experiments and sampling. WVdB participated in the analysis and the interpretation of all the morphological results. SG took part in the design of the study and critically analyzed all the results. HJN coordinated the study and participated in its design. All authors read and approved the final manuscript.
